# Hyperparasitaemia and low dosing are an important source of anti-malarial drug resistance

**DOI:** 10.1186/1475-2875-8-253

**Published:** 2009-11-11

**Authors:** Nicholas J White, Wirichada Pongtavornpinyo, Richard J Maude, Sompob Saralamba, Ricardo Aguas, Kasia Stepniewska, Sue J Lee, Arjen M Dondorp, Lisa J White, Nicholas PJ Day

**Affiliations:** 1 Mahidol - Oxford Tropical Medicine Research Unit, Faculty of Tropical Medicine, Mahidol University, 420/6 Rajvithi Road, Bangkok 10400, Thailand; 2 Centre for Tropical Medicine, Nuffield Department of Clinical Medicine, University of Oxford, Oxford, UK; 3 Instituto Gulbenkian de Ciência, Oeiras, Portugal

## Abstract

**Background:**

Preventing the emergence of anti-malarial drug resistance is critical for the success of current malaria elimination efforts. Prevention strategies have focused predominantly on qualitative factors, such as choice of drugs, use of combinations and deployment of multiple first-line treatments. The importance of anti-malarial treatment dosing has been underappreciated. Treatment recommendations are often for the lowest doses that produce "satisfactory" results.

**Methods:**

The probability of de-novo resistant malaria parasites surviving and transmitting depends on the relationship between their degree of resistance and the blood concentration profiles of the anti-malarial drug to which they are exposed. The conditions required for the in-vivo selection of de-novo emergent resistant malaria parasites were examined and relative probabilities assessed.

**Results:**

Recrudescence is essential for the transmission of de-novo resistance. For rapidly eliminated anti-malarials high-grade resistance can arise from a single drug exposure, but low-grade resistance can arise only from repeated inadequate treatments. Resistance to artemisinins is, therefore, unlikely to emerge with single drug exposures. Hyperparasitaemic patients are an important source of de-novo anti-malarial drug resistance. Their parasite populations are larger, their control of the infection insufficient, and their rates of recrudescence following anti-malarial treatment are high. As use of substandard drugs, poor adherence, unusual pharmacokinetics, and inadequate immune responses are host characteristics, likely to pertain to each recurrence of infection, a small subgroup of patients provides the particular circumstances conducive to de-novo resistance selection and transmission.

**Conclusion:**

Current dosing recommendations provide a resistance selection opportunity in those patients with low drug levels and high parasite burdens (often children or pregnant women). Patients with hyperparasitaemia who receive outpatient treatments provide the greatest risk of selecting de-novo resistant parasites. This emphasizes the importance of ensuring that only quality-assured anti-malarial combinations are used, that treatment doses are optimized on the basis of pharmacodynamic and pharmacokinetic assessments in the target populations, and that patients with heavy parasite burdens are identified and receive sufficient treatment to prevent recrudescence.

## Background

Resistance to anti-malarial drugs poses a major threat to malaria control and elimination. Anti-malarial drug resistance emerges de-novo when malaria parasites with spontaneously arising mutations or gene duplications conferring reduced drug susceptibility are selected by anti-malarial drug concentrations sufficient to suppress the growth of sensitive, but not the newly arisen resistant mutant parasites [1-4]. For these new resistant parasites to spread to other hosts, the resistance mechanism must not affect their fitness greatly, so that the resistant parasites can expand in numbers to generate gametocyte densities sufficient for transmission to biting anopheline mosquitoes [5]. As the de-novo resistance event is probably independent of the drug effect it can happen whenever there is DNA replication. It could arise in the vector mosquito (where meiosis occurs), during the pre-erythrocytic liver stage development, or during the blood stage infection [6]. There has been much debate and controversy over the likely source of de-novo anti-malarial resistance and its geographic origins. The numbers of malaria parasites circulating in areas of high malaria transmission are considerably greater than in areas of low and seasonal transmission, and so early predictions were that resistance would arise more frequently in these areas [7]. History indicates the opposite. Resistance to the main anti-malarials chloroquine, sulphadoxine-pyrimethamine, mefloquine, and artemisinin, has arisen in low transmission areas and then spread [8]. South East Asia has been a consistent epicentre of resistance. Resistant parasites originating there have spread to Africa. In contrast, the emergence and spread of anti-malarial drug resistance seems to have been slowest in areas of high stable transmission. The principle reasons for this difference is the considerable brake on resistance emergence and spread conferred by host immunity, and the associated large transmission reservoir provided by asymptomatic untreated individuals, which dilutes the selective pressure provided by the anti-malarial drugs. Mathematical modelling of anti-malarial resistance has tended to focus on parasitological factors and simplify host contributions to the emergence of resistance. Here, the importance of anti-malarial dosing, and the particular role that patients with heavy parasite burdens play in generating anti-malarial drug resistance, and the circumstances most conducive to its subsequent spread are examined.

### Intra-host malaria population dynamics

Heritable anti-malarial drug resistance could arise at any nuclear division. Within host parasite numbers vary in the course of a malaria infection over six to 12 orders of magnitude. After sporozoites are inoculated by a feeding female anopheline mosquito they find their way to the liver within one hour. Each infects a hepatocyte. In human malarias the actual numbers inoculated are not known, but indirect studies suggest a skew distribution with a median value of approximately 8-10 sporozoites [3,4]. Multiplication within the entire human infection is asexual with replication by mitosis. Within these few infected liver cells the parasites divide repeatedly every eight hours or so for approximately 16 serial sets of divisions until each cell contains some 35,000 merozoites. After approximately 5.5 days in *Plasmodium falciparum *infections the infected liver cells burst (schizont rupture) liberating the infectious merozoites into the blood stream. The merozoites rapidly invade passing erythrocytes. This is the number of parasites that would be exposed to anti-malarial drugs when a newly acquired infection encounters residual anti-malarial drug levels from a previous treatment, or during chemoprophylaxis. For *Plasmodium falciparum, Plasmodium vivax*, and *Plasmodium ovale*, each asexual cycle within the red cells lasts approximately two days and multiplication initially is usually reasonably efficient (20-50% efficiency). The average parasite multiplication factor (PMF) per generation in non-immunes usually ranges between 5 and 25 with estimated median values of 8 to 13 [9-11]. This results in exponential population growth. Multiplication factors drop abruptly soon after the patient becomes ill. Numerous factors, not all of which are understood, contribute to this abrupt reduction in PMF. These include activation of non-specific host defence mechanisms, increased splenic clearance function, inhibition of schizont development by fever, and exhaustion of susceptible erythrocytes. Quorum sensing in malaria parasites has not been well characterized. Without anti-malarial treatment the intra-host parasite population usually stabilizes and then eventually declines [9-14]. Hyperparasitaemia and a fatal outcome can be considered an unusual failure of these various host and parasite control mechanisms. A parasite burden of 5 × 10^11 ^parasites in an adult gives a parasitaemia of approximately 1% (depending on parasite stage and synchronicity of infection). This is not yet a lethal burden, but the next multiplication step is critical to outcome. The patient is teetering on the edge of a precipice as a PMF ≥ 5 will create a potentially lethal sequestered biomass [15], whereas a factor of one or less will almost guarantee recovery. Effective anti-malarial treatment reduces multiplication abruptly resulting in a rapid decline in parasite numbers. This is a first order process, resulting in killing of a fixed fraction of the parasite population each asexual cycle. Fractional reductions in parasite numbers per asexual cycle (parasite reduction ratios [PRR] which are the reciprocal of the PMF) are typically 1000, or with artemisinin treatment as high as 10,000 [16].

Anti-malarial drug treatment limits transmissibility of the infection as the sexual stages derive from the asexual stages, which are killed by the drugs. Peak gametocyte densities are therefore a function of peak asexual parasite densities. Falciparum malaria differs from the other human malarias in that gametocyte production is delayed with respect to asexual parasite multiplication; peak gametocytaemia occurs seven to10 days later [5,12,17]. As a result effective treatment has a greater effect in reducing the transmissibility of *P. falciparum *infections compared with the other human malarias.

### The relationship between the level of anti-malarial resistance and the probability of selecting resistance de-novo

Drug resistance describes a right shift, and sometimes a change in shape, of the concentration-effect relationship. Evaluations of anti-malarial resistance have tended to consider resistance as a binary (or sometimes ternary) variable, whereas there is more commonly either a continuous or multimodal distribution of susceptibility to any drug. It is important to note that resistance is not equivalent to treatment failure. Patients with fully sensitive parasites may fail treatment because of inadequate treatment or pharmacokinetic factors, and patients with highly resistant parasites may be cured because of the host's immunity. The *degree of resistance *conferred by a genetic event in malaria parasites is critically important both to therapeutic response and to the probability of the resistant parasites being selected. There is no selection of resistance if the anti-malarial drug concentrations are high enough to kill all the sensitive and all the resistant parasites, or too low to kill either. Thus, there is a window of concentrations for any particular level of resistance that provide a selection opportunity [18].

De-novo anti-malarial drug resistance is relevant at a population level only if it is transmitted to other people. This requires that the resistant parasites expand in numbers and produce sufficient gametocytes to infect an anopheline mosquito vector. If the resistance mechanism conveys a small increment in resistance, as most mechanisms do initially, then it offers limited opportunity for selection i.e. provision of the difference in growth rates necessary for the resistant sub-population to expand with respect to the drug-sensitive sibling population [18]. On the other hand, if the new genetic event confers a large reduction in susceptibility in relation to therapeutic drug concentrations, either upon a fully sensitive "wild-type" population (e.g. the *cytochrome b *mutations which confer atovaquone resistance), or upon an already resistant parasite population (e.g. the *Pfdhfr *I164L mutation, which invariably occurs in parasites with other resistance mutations in this gene), then de-novo selection occurs readily. This is because "high-grade" resistance allows survival of parasites within the range of anti-malarial drug concentrations that follow a standard dose ("therapeutic concentrations"), whereas low grade resistance does not. This increases the survival probabilities for resistant mutants considerably, and it creates the conditions necessary for selection. Thus the de-novo selection probability is directly proportional to the level of resistance produced by the genetic mutation or amplification. As a result, if anti-malarial resistance progresses in a stepwise fashion (such as antifol or sulpha resistance), and other factors are equal, the rate of development of resistance would be expected to accelerate. This is because with increasing levels of resistance a greater proportion of de-novo resistant parasites (and their progeny) can survive in the anti-malarial drug concentrations that follow standard dosing.

Recrudescence of the infection is essential for transmission of de-novo resistant parasites. This is because the new resistant parasites need to multiply from low numbers (commonly only one) to reach a density sufficient to generate enough gametocytes to be taken up by a feeding mosquito (a human body total of approximately one hundred million parasites). At the same time the multiplication of drug-sensitive sibling parasites must be suppressed. For a two-day asexual cycle, at a PMF of 10, this process takes 16 days.

### Assessing levels of resistance

The level of resistance can be characterized in-vivo by the PRR values of the resistant parasites in the anti-malarial blood concentration range, which occurs during and following treatment. The PRR per cycle ranges from 0.1 (no drug effect) to >10,000 with maximum effects from artemisinin derivatives [16]. The lowest anti-malarial concentration resulting in the maximum possible PRR is the minimum parasiticidal concentration (MPC), and the concentration producing a growth rate of 1 is the minimum inhibitory concentration (MIC). By definition resistant parasites have higher MICs and MPCs than sensitive parasites. Unfortunately, the relationship between these in-vivo measures, and conventional in-vitro measures of susceptibility (IC_50 _or IC_90_) is not well characterized. This is because conventional tests measure inhibition of growth (but not multiplication) under constant drug exposure in conditions very different to that in-vivo.

**Figure 1:**
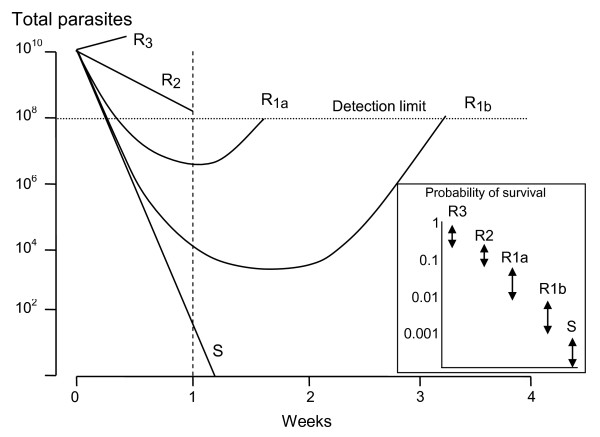
**Different levels of anti-malarial drug resistance provide different probabilities of individual parasite survival (inset)**. The total numbers of parasites in an individual patient change with time. Treatment failure is defined as a failure to clear detectable parasites from the blood (R3, R2) or a subsequent recrudescence (R1). The former WHO classification of resistance translates into growth rates per cycle (PMF) as follows; R3; >0.25, R2; 0.1 to 0.25, R1a; 0.1 to 0.01, and R1b; <0.01. The inset shows the corresponding probabilities of an individual parasite surviving one cycle if exposed to therapeutic drug concentrations.

There is also often considerable variation between laboratories in in-vitro susceptibility assessments. In the early stages of drug resistance, in which there is low grade resistance, the concentrations of anti-malarial drug that immediately follow treatment still exceed the MPC and it is only after parasite densities have fallen below the level of detection that levels fall below the MIC. There is no prolongation of parasite clearance times. As resistance worsens (i.e. the MPC rises) blood concentrations fall below the MPC before parasitaemia falls below the level of detection. Only then do parasite clearance times begin to lengthen. Eventually, with further increases in resistance, parasite reduction is insufficient to clear parasitaemia within seven days (Figure 1).

The old WHO classification of resistance provides a convenient stratification for levels of resistance observed in in-vivo drug trials, which can be translated readily into PRR values.

R3: the highest level of resistance in which parasite density falls by less than 75% in 48 hours (one cycle). This results from a PRR of < 4/cycle.

R2: Parasitaemia falls by more than 75% in 48 hours, but fails to clear in 7 days; this results from a PRR of between 4 and 10/cycle, assuming an upper limit of the total body parasite burden in clinical trials of 10^12 ^parasites/person and a parasite detection limit of 10^8 ^parasites/person.

R1: recrudescence after seven days; assuming an upper limit of the total body parasite burden in clinical trials of 10^12 ^parasites, this requires a PRR >10/cycle. This can be further subdivided into R1a in which parasitaemia does not clear within 96 hours (PRR 10-100), and those infections in which it does (PRR>100/cycle).

The corresponding growth rates per cycle are therefore; R3; >0.25, R2; 0.1 to 0.25, R1a; 0.1 to 0.01, and R1b; <0.01 (Figure 1). These are population averages but can be translated into individual parasite probabilities of survival and indicate that individual merozoite probabilities of survival vary over almost five orders of magnitude. Thus for a similar probability of mutation resistance is up to 10,000 times more likely to emerge for very high grade resistance than for the lowest grade of resistance.

### Implications

1. There is a selective window of anti-malarial drug concentrations within which any given level of resistance can emerge de-novo.

2. High grade resistance is selected more readily than low grade resistance

3. If resistance develops in a series of equal increments, the rate of emergence increases.

### Quantitating the risks of selecting and transmitting resistance

Malaria parasites enjoy a brief moment of diploidy in the mosquito vector, but thereafter have a haploid existence. Numbers vary from as few as the inoculation of a single sporozoite to nearly 10^13 ^blood stage asexual parasites in a hyperparasitaemic adult. The emergence of drug resistance in malaria parasites in relation to their site and stage of development has been modelled recently [6]. These probabilities are examined in Additional file 1.

#### Parasite multiplication efficiency and parasite burdens

For resistance arising de-novo during the blood stage infection the probability distribution for numbers of resistant parasites present in the body is a function of the multiplication rate per asexual cycle, and the total number of parasites. PMF can range from negative values (with self-cure, or following anti-malarial drug treatment) to nearly 30 per asexual cycle. The absolute upper limit is set by the number of merozoites per schizont. Parasite multiplication is always inefficient. A maximum (growth phase) multiplication rate of 20 is very high and represents greater than 50% efficiency (E> 0.5), whereas a multiplication rate of two represents approximately 6% efficiency (E = 0.06). Such a low multiplication rate probably could not be sustained for long enough to generate high parasite burdens because of the induction of density controlling non-specific and specific host-defence mechanisms. For example it would take approximately one month from hepatic schizogony for an infection with a PMF of 2 to reach a detectable parasitaemia. Furthermore with low multiplication factors, even if a resistance mutation arose during mitosis, the resulting resistant merozoite would have a correspondingly low probability of invading and replicating successfully, whereas with highly efficient multiplication these probabilities might exceed 50%. It is important to note that the multiplication of parasite populations which have already reached high densities is very unlikely to be efficient (and if they were, the outcomes would probably be fatal, and therefore these patients could not transmit de-novo resistance). This large reduction in E at high densities has an important impact on the survival of any de-novo resistant parasite emerging in the multiplication step that generated the high densities irrespective of drug effects. For example if E falls ten fold resulting in a drop in PMF from 10 in growth phase to one at peak parasitaemia then the probability of there being 10 (i.e. from the previous cycle) and one (current cycle) potentially viable de-novo resistant intra-erythrocytic parasites becomes equal.

**Figure 2:**
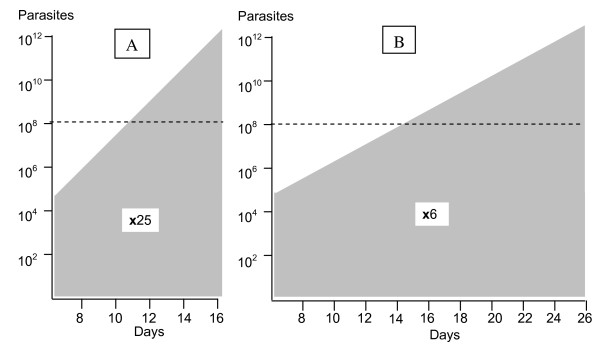
**Total numbers of malaria parasites in the body of two adults (logarithmic scale; vertical axis) who develop similar hyperparasitaemias are shown**. The days since acquiring the infection are shown on the horizontal axis. The pre-erythrocytic development is approximately 6 days. On the left A; the parasite multiplication factor (PMF) per asexual cycle is 25 representing highly efficient multiplication. On the right B; the multiplication factor is 6 per cycle, so it takes longer to develop hyperparasitaemia.

In non-immune patients multiplication factors for *P. falciparum *in the growth phase of the infection can be approximated to 10 per two-day asexual cycle [10,11], representing 30-40% multiplication efficiency (E = 0.3 to 0.4; additional file; equation 3). Patients with acute malaria present a range of parasite multiplication histories. Some will have been ill for many days with a relatively low multiplication factors, significant red cell loss, and consequent progressive anaemia. Others will have a short history with highly efficient parasite multiplication and often little or no anaemia (Figure 2). Hyperparasitaemia probably represents the more efficient multiplication end of the spectrum, as low multiplication factors reflect either host immunity or a resistant host phenotype and are unlikely to be sustained for long enough to develop hyperparasitaemia. This explains why hyperparasitaemic non-immune patients are often not anaemic (as the degree of anaemia is proportional to the duration and severity of the infection). In higher transmission settings the dynamics are more complex.

Doubling the multiplication rate doubles the efficiency of replication and therefore doubles the probability that a de-novo resistant parasite would survive. A rapidly expanding infection is therefore more likely to generate viable resistant parasites, and is also more likely to reach high densities before multiplication factors fall (Figure 2A). But if hyperparasitaemia did arise in a slowly expanding infection (Figure 2B), it would have arisen as a result of a greater number of cell divisions, and have a correspondingly greater probability of there being more than one resistant mutant parasite present. This is outweighed by the reduced survival probabilities that gave the low multiplication rate in the first place.

#### Initial anti-malarial drug effects

Drug effects reduce the basal parasite multiplication rate. Anti-malarial drugs act mainly at middle of the asexual cycle, and have relatively less effects on formed schizonts. Drug exposure occurs at peak parasitaemia. As the most likely series of mitotic divisions to give rise to resistance is the most recent (i.e. the mitotic events that gave rise to the peak parasitaemia), the modal number of resistant merozoites produced is one. But the survival probability of this usually single parasite is low, as even if there was no treatment, continued efficient multiplication is very unlikely. For example a PMF of approximately one, which would be quite likely in the absence of treatment at high parasite densities, provides a probability of 7.5% of successful progress of a resistant merozoite to the next asexual cycle. This assumes an absolute maximum of 32 merozoites per schizont, although it may be more realistic to assume this maximum is 16 -thereby doubling the probabilities (Additional file 1, equation 5). Thus, only one in 14 de-novo events at this stage could progress one cycle even if the level of resistance it conferred was so high that the peak anti-malarial blood concentrations did not reach the MIC_R_. If the resistant parasite's progeny does survive one cycle-at least one of the merozoites produced by the subsequent "de-novo resistant" schizont 48 hours later must also survive and invade successfully. This requires that the anti-malarial blood concentrations 48 hours after starting treatment must still not exceed MIC_R_. For most resistance mechanisms the initial levels of de-novo resistance are insufficient for malaria parasites to survive therapeutic blood concentrations of the anti-malarial drug. In other words these de-novo resistant parasites are still killed by "therapeutic concentrations". This is why incomplete treatment is used in experimental models for selecting resistance - and why incomplete treatment is so dangerous in clinical practice. During the earlier growth phase of the infection the chances of individual parasite survival are up to ten times greater than at peak parasitaemia, but as overall parasite numbers are less, opportunities for resistance to emerge are between 10 and 10^7 ^times lower. Even during the most efficient growth phase only high levels of resistance can survive therapeutic drug concentrations.

Peak anti-malarial drug concentrations are usually log-normally distributed. If low drug concentrations occur because of poor adherence, vomiting, malabsorption, sub-standard drugs, or unusual pharmacokinetics, then survival probabilities for resistant parasites increase. The increase is non-linear reflecting the shape of the shifted dose response curve and the shape of the probability distribution of drug concentrations. Resistance may be selected if there is a sufficient difference in drug susceptibilities between resistant and sensitive parasites (MIC_R _minus MIC_S_) as discussed below.

#### Growth competition between resistant and sensitive parasites

De-novo resistant parasites can be considered in growth competition with their drug sensitive, but otherwise identical, sibling parasites. This is particularly relevant to the possible selection of artemisinin resistance as discussed later. The width of the anti-malarial drug concentration window of selection within which selection can take place, is proportional to the degree of resistance [18]. If anti-malarial plasma concentrations are below the window (underdosing, sub-standard drug, poor adherence, vomiting, etc) then there will not be enough suppression of the sensitive parasites, which will outgrow their resistant siblings, and beat them in the race to reach transmissible gametocyte densities. At a PMF of 10/cycle it takes one resistant parasite over two weeks to generate enough progeny to transmit. Recrudescence is necessary for selection (i.e. suppression of sensitive and expansion of resistant parasites), and these recrudescent infections have a greater per-infection probability of patent gametocytaemia than primary infections. Recrudescent infections are, therefore, the source of resistance spread [19]. This is examined in more detail below.

**Figure 3:**
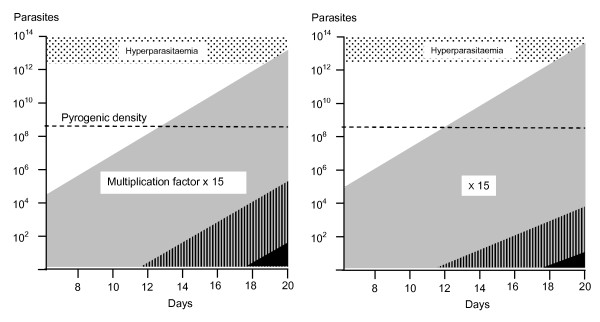
**Total numbers of malaria parasites in the bodies of two adults (logarithmic scale; vertical axis) with similar rapidly expanding infections who develop hyperparasitaemia (>5% parasitaemia) are shown against time since their infecting mosquito bites on the horizontal axis**. The horizontal dotted line is the pyrogenic density (the density threshold for fever). The most likely mitotic division to give rise to de-novo resistance is the one immediately preceding peak parasitaemia. The most likely (i.e. modal) number of resistant parasites present therefore is one. There are correspondingly lower probabilities of there being much larger numbers of de-novo resistant parasites. The inset triangles represent the much rarer situation of earlier de-novo appearance of resistant mutants and the corresponding greater numbers of resistant parasites that would occur at presentation with hyperparasitaemia. In the example on the left the fitness and thus multiplication factor of the mutant parasites is unaffected by the resistance mechanism, whereas on the right their fitness and thus multiplication is reduced relative to their drug sensitive siblings.

Before anti-malarial drug exposure multiplication rates of the resistant parasite(s) will be lower (if fitness <1) than or equal to the sensitive parasites. As discussed above if resistance does arise de-novo then the modal number of resistant mutant merozoites will be one, but there is a smaller probability (determined by the multiplication rate) that resistance arose earlier in the infection i.e. in an earlier asexual generation or during liver stage development (i.e. before treatment; Figure 3) and so generated larger numbers of progeny. The probability that there is more than one resistant parasite present at the time of first drug exposure is obviously greater in patients with a high parasitaemia. For resistance to spread the progeny of these parasites must survive, and eventually expand in numbers sufficient to generate enough gametocytes to transmit.

Usually resistance is associated with reduced fitness, and thus reduced multiplication in comparison with drug sensitive siblings. If this is the case, then the newly arisen resistant mutants will comprise a smaller proportion of the total parasite numbers at presentation than they did at the time of their origin (Figure 3, right side). If there is only one resistant parasite present (initially as a merozoite) then the only anti-malarial concentrations that can be selective are equal to or less than the minimum inhibitory concentration (MIC_R_) [16]. This is because for the single parasite's progeny to survive it must obviously have a multiplication factor of ≥1 [18]. For larger numbers of parasites arising from an earlier resistance event, anti-malarial concentrations can exceed the MIC temporarily, as long as they fall to ≤ MIC before elimination of all the resistant parasites. This allows emergence of a lower grade of resistance.

In summary, the rare instances when de-novo resistance arises very early in the infection are particularly conducive to resistance emergence for two reasons:

1. The total number of resistant parasites exposed to the anti-malarial drug is greater, and the resistant population may, therefore, survive temporarily at concentrations above the MIC for the resistant parasites (MIC_R_).

2. The sensitive parasite population is correspondingly reduced providing less growth competition.

For example, if anti-malarial drug resistance developed in the mosquito and half the inoculated sporozoites were resistant, then if there were no fitness disadvantage, half the blood stage parasites at any time before drug exposure would be resistant [6].

#### Failure of host defence mechanisms and hyperparasitaemia

**Figure 4:**
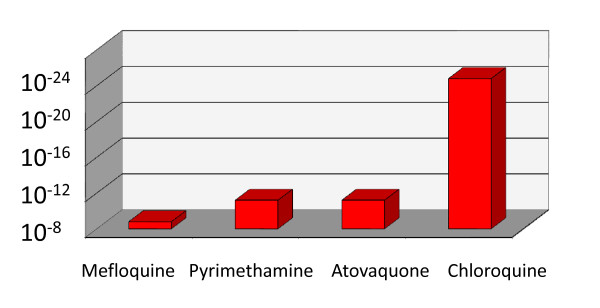
**Approximate per-parasite probabilities of de-novo emergence of viable and transmissible resistant parasites in clinical infections**. Mefloquine resistance results from *Pfmdr *amplification, pyrimethamine resistance from mutations in *Pfdhfr*, atovaquone resistance from mutations in *Pfcyt b*, and resistance to chloroquine from mutations in *PfCRT*.

Immunity provides a powerful obstacle to the emergence of resistance as immune responses kill parasites irrespective of their level of drug resistance. Anti-malarial drug resistance historically has arisen predominantly in areas of low malaria transmission where individuals have little or no immunity. In endemic areas infants and young children are relatively non-immune. Unless effective treatment is readily available a greater proportion of infections in non-immune patients reach high densities. Patients with a high parasitaemia have, by definition, failed to control their infection. The failure of host defence, which allowed unrestricted parasite multiplication and consequent hyperparasitaemia reflects several processes, which facilitate the emergence of resistance. Evidently there is little or no effective specific immunity against these particular parasites, even if there is against others, and non-specific defences (splenic activation, innate immunity etc) have been inadequate to control the parasite population expansion. The higher recrudescence rates following anti-malarial treatment of hyperparasitaemic infections results from this failure of host defence, and that more treatment is required to eliminate high burdens. It has been suggested that the *P. falciparum *variant surface antigen specific antibody response would reduce considerably the emergence of resistance. The volunteer experiments of Martin and Arnold and others in which high level pyrimethamine resistance in *P. falciparum *was selected readily and repeatedly in non-immune volunteers, and was more likely to emerge in those with a higher parasitaemia receiving a low dose, suggest this is not a major impediment [20,21] (Table 1). The selection of atovaquone resistance in one third of non-immune acute falciparum malaria recipients also indicates the ease with which high-level resistance can emerge under drug selection in-vivo in a single passage [22]. The amplification in the *Pfmdr1 *gene, which causes mefloquine resistance, occurs very frequently and emerges at an estimated frequency of 1 in 10^8 ^mitotic divisions. It, therefore, develops frequently within an infection [23,24] (Figure 4). Rapid selection of *Pfmdr1 *amplification is balanced by the considerable fitness cost, so resistance is lost rapidly when drug selective pressure is lifted.

**Table 1:** The rapid selection of pyrimethamine resistance in-vivo; recrudescence proportions in volunteers with falciparum malaria treated with pyrimethamine; from Martin and Arnold 1968 (21).

**Pyrimethamine dose (mg)**	**Highest observed parasite count/uL**				
	**<1000**	**1000 to 20,000**	**20,000 to 100,000**	**100,000 to 200,000**	**>200,000**
12.5	2/5	1/1			
25	0/10	5/5			
50			2/2		
100		0/1	0/1	0/1	1/1
150				0/1	

Volunteers were given the Ugandan strain of *P. falciparum *by intravenous injection and treated with a single dose of pyrimethamine. All recrudescences occurred within 21 days. Recrudescent infections were highly resistant to treatment with 150 mg of pyrimethamine. The parasites were obtained from a child in Kampala in 1965. Pyrimethamine had been used extensively over the previous ten years, but not apparently in the area where this child originated from. Nevertheless the parasites may already have had some degree of resistance (i.e. resistance mutations in *Pfdhfr*).

#### Critical role of treatment failure in the genesis and transmission of resistance

Treatment failure leading to recrudescence is required in nearly all cases for the transmission of de-novo resistance. Any gametocytes transmitted from the acute illness episode from which the new resistant parasites arose (the primary infection) will derive from the much larger pool of sensitive asexual parasites - except for the very rare event when resistance emerged very early in the infection [5,25]. The primary infection gametocytes are, therefore, very unlikely to contain any de-novo resistant mutants. For example, if it is assumed arbitrarily that at least 10% of the gametocytes must carry the new resistance mutation for resistance to be transmitted [18], then with a parasite multiplication factor of 10, at least 10% of the parasites emerging from the liver must carry the resistance mechanism for transmission of resistance to occur from the primary infection. This leads to an important conclusion. Transmission of de-novo resistance from the primary infection cannot occur unless the resistance mechanism arose in the liver stages.

Except in those very rare cases [6], which are approximately one million times less frequent than emergence during the blood stage of a hyperparasitaemic infection, the gametocytes, which transmit resistance, will arise from the subsequent recrudescent infection. This requires suppression of the sensitive parasites by drug treatment so that only the resistant parasites can multiply and produce the gametocytes in the recrudescent infection [25]. This is an important bottleneck [6]. It is also evident that if malaria parasites have already acquired high-grade resistance resulting in early treatment failures -further selection does not occur, so emergence of an even higher grade of resistance in this context is not possible. For example, the reason that *P. vivax *has not acquired an mutation analagous to the *Pfdhfr *I164L mutation in *P. falciparum *(conferring high grade antifol resistance) in South-East Asia may be because the other *Pvdhfr *and *Pvdhps *mutations it has developed there provide such high grade resistance to sulphadoxine-pyrimethamine, that further selection is unlikely [26].

As described above the risks of treatment failure increase with increasing pre-treatment parasite counts [27-31]. For example on the north-western border of Thailand failure rates with the three-day regimen of artesunate and mefloquine approximate 5%, but if the patient has >4% parasitaemia the failure rate approximates 30% [32]. Multiple treatments may be required for the selection of resistance, as is often the case in animal models, and so the much higher rate of treatment failure with hyperparasitaemia, and the higher rate of further treatment failure in recrudescent infections conspire to create a scenario conducive to selection. This scenario is more likely in low transmission settings where the immune response to malaria is not primed, and multiple recrudescences commonly follow a first recrudescence.

#### Asymptomatic parasitaemia

In malaria endemic areas, most people are infected with malaria parasites most of the time, and many adults and older children harbour a detectable parasitaemia for long periods. These malaria parasites survive by evading the immune response through antigenic variation and competing successfully with other malaria parasites. These infections intermittently produce transmissible densities of gametocytes. *Plasmodium falciparum *asexual parasite densities up to 10,000/uL can be asymptomatic. As individuals with asymptomatic parasitaemia may take anti-malarial treatments for fevers (unrelated to malaria) they do provide a resistance selection opportunity. Three factors reduce the probabilities of selection in asymptomatic parasitaemia compared to that in symptomatic infections:

1. *The effectiveness of the immune response that controlled parasite densities below pyrogenic levels*.

This prevents emergence from the main parasite population and requires the de-novo resistant parasite to derive from a new antigenically variant and poorly recognized sub-population if the high growth rates necessary to sustain low-grade resistance, and produce sufficient gametocyte densities are to occur. The effects of any fitness disadvantage are amplified at low overall parasite growth rates.

2. *The low parasite density, and thus low number of mitoses*.

In a symptomatic infection anti-malarial treatment is sought and taken exposing between 10^7 ^and 10^13 ^malaria parasites to the drugs used. In an asymptomatic infection parasite numbers are below the pyrogenic density. If anti-malarial treatment is taken for another incidental febrile infection it exposes between 10^4 ^and 10^10 ^malaria parasites to the drugs used. The asymptomatic infection, therefore, presents a much lower probability of large numbers of de-novo resistant parasites being present because total parasite numbers are usually orders of magnitude lower, and also because growth of the resistant sub-population is required to generate transmissible gametocyte densities.

3. *Growth kinetics*.

In contrast to a symptomatic malaria infection, which has expanded rapidly to produce pyrogenic densities of malaria parasites in the blood, and is then treated, an asymptomatic infection is much less likely to be in growth phase. Each asymptomatic infection parasite population is in one of three phases (growth, quasi-steady state, or elimination), whereas all symptomatic infections have just been or are in growth phase. There is insufficient information on the kinetic properties of these phases in asymptomatic infections, but it is obvious that each individual parasite inoculation cannot individually reach detectable parasite densities and then persist otherwise everyone living in endemic areas would have high density superinfections. The most likely scenario is a series of superimposed waves of parasitaemia with different amplitudes and frequencies, but overall cumulative densities controlled below pyrogenic levels (<10,000/uL) [33]. This control is a complex process but comprises, in part, immune responses which will be directed equally at sensitive and resistant parasites.

The processes which suppress expansion of the parasite population are also likely to control multiplication of any drug resistant mutants. Thus, the survival probability of any newly formed resistant parasites is greatest during the growth phase before immune control intervenes. The lower the overall multiplication rate the lower is the probability of resistant parasites reaching transmissible densities, both because of immune control and the relatively greater effects of any fitness disadvantage in competitive multiplication.

### Implications

1. Resistance is more likely to arise with high parasite growth rates and high parasite burdens.

2. There is a complex trade-off in the probabilities of a de-novo resistant parasite surviving between multiplication efficiencies, parasite numbers, drug concentrations and drug effects.

3. In nearly all circumstances, recrudescences, and in most cases, multiple recrudescences are required for the de-novo selection of resistance.

4. Once high-level resistance is established, even higher levels cannot be selected.

5. Inadvertent anti-malarial treatment of an asymptomatic parasitaemia is an unlikely source of de-novo resistance.

### Pharmacokinetic properties of relevance to de-novo selection

For resistance to spread, anti-malarial drug exposure must be sufficient to suppress the growth of sensitive parasites so that they comprise <90% of the total at the time of recrudescence. This requires that the treatment provides a sufficient differential effect [18]. High-level resistance confers a large differential between the inhibitory effects on sensitive parasites and the resistant sub-population, and so, once de-novo high grade resistance has emerged, it is more likely to survive (e.g. atovaquone resistance) [22]. If not all the drug sensitive sibling parasites are eliminated and some survive then they will compete with the newly arisen resistant parasites. Competition from drug-sensitive siblings is particularly important for rapidly eliminated drugs. Slowly eliminated drugs provide two or more successive asexual cycles of differential growth suppression (i.e. selection). Each cycle during which there is some suppression of sensitive parasite growth increases the difference between the newly emergent resistant and the sibling sensitive parasites and thus the chances of selection and ultimately transmission. This explains why, under some circumstances, resistance emerges more readily to slowly eliminated drugs [34], although slow elimination is of much greater relevance to the spread of resistance as it provides a selective filter for resistant parasites acquired from elsewhere. As resistance is usually associated with a fitness disadvantage in competitive growth, it may be necessary to eliminate all the sensitive parasites for the resistant mutants to survive.

**Figure 5:**
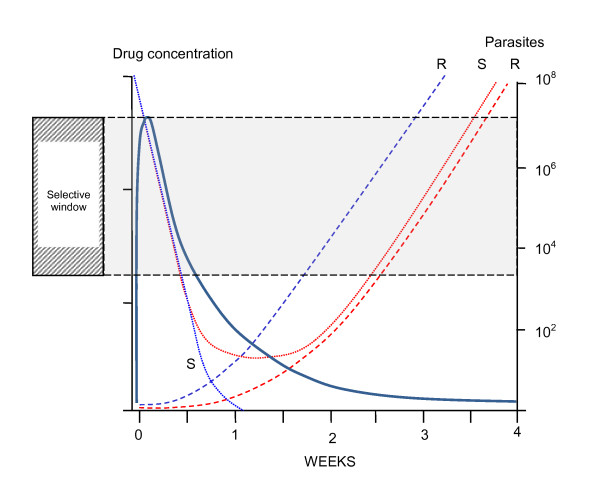
**The anti-malarial concentration window of selection **[18]. The drug concentrations (solid lines) following two doses which define the concentration limits of the window of selection are shown for patients with low parasitaemias (10^8^/body). Total parasites within the body are depicted by dotted and dashed lines. The window opens when following the higher dose (blue) sensitive parasites (blue dotted line) are eliminated but de-novo resistant parasites (blue dashed line) can survive, and successful selection of resistance can take place. Following the lower dose the sensitive parasites can still survive (red dotted line) and later grow to outnumber the drug resistant parasites (blue dashed line) by >9:1, so there is no longer an opportunity for preferential survival of the resistant parasites and thus selection. The window closes. Concentrations between these two boundaries (the "window of selection") are selective.

The relationship between terminal elimination half-life of the anti-malarial drug and opportunities for resistance selection from infections newly acquired following drug administration has been analysed in terms of a "window of selection" [18]. The window was defined as the period of time (containing a gradient of drug concentrations) during which resistant parasites had the opportunity to multiply and comprise ≥10% of gametocytes. The mathematical details are given in Additional file 2. A similar approach can be taken to initial anti-malarial drug concentrations in that there is a window of concentrations within which selection of a particular level of resistance can take place (Figure 5). The upper limit of the window is defined by the plasma concentration of drug which will kill both sensitive and resistant parasites. These have been defined in terms of probabilities, which decline asymptotically so the limit can be set at an arbitrary level (such as less than 1% probability of survival). The lower limit is defined by the plasma concentration at which the inhibition of sibling sensitive parasites' growth is insufficient to prevent their progeny comprising ≥ 90% of the gametocytes. Thus, there are a series of windows for different numbers of de-novo resistant parasites. The width of the window is determined by the degree of resistance i.e. the difference in drug susceptibility between the de-novo resistant mutant(s) and the remainder of the more sensitive parasites. The majority parasites may already be partially resistant, such as the stepwise acquisition of antifol or sulpha resistance. This narrows the window. Resistance arising in an earlier generation resulting in large numbers of resistant parasites before drug exposure widens the window. If the de-novo resistance mechanism confers a large decrease in drug susceptibility then the window is wide, and may involve therapeutic concentrations of the drug (e.g. e.g *Pfdhfr *164 mutation; high-grade pyrimethamine resistance) or narrow such that only sub-therapeutic concentrations can select (e.g *Pfdhfr *108 mutation; low-grade pyrimethamine resistance). Anti-malarial drug plasma concentration profiles vary considerably between patients. The closer anti-malarial drug concentrations are to the range giving sub-maximal effects in all patients (with sensitive parasites), the higher is the probability that some patients have sub-therapeutic levels and so there is a greater opportunity for selection in the narrow window of selection for low-grade resistance. This happens with systematic underdosing (e.g. sulphadoxine-pyrimethamine treatment regimens in children). It is a strong argument for recommending dose regimens which guarantee maximum effects in nearly all patients infected with sensitive parasites. These regimens will provide a greater safety margin (in terms of efficacy) and thereby reduce the selection opportunities for low-grade resistance. The concept of mutation prevention in bacterial infections through dose optimisation is now well established [35]. Similar arguments apply to anti-malarials. If low-grade resistance is a necessary stepping-stone to higher levels of resistance, then dose optimization [36] together with the use of combination treatments, provide the best opportunity to prevent the emergence of resistance.

The pharmacokinetic properties of anti-malarial drugs vary considerably between individuals. At the extreme end of the distribution lie patients with very low anti-malarial drug concentrations despite having taken and retained a full treatment dose. These patients are obviously more likely to fail treatment, and they are also more likely to fail again when retreated, as they will probably have low drug levels again.

### Implications

To prevent the emergence of resistance dose regimens should aim to provide therapeutic concentrations in nearly all patients. Current anti-malarial dose regimens tend to be too low.

### Characteristics associated with hyperparasitaemia of relevance to resistance

#### Parasite burdens

A high parasitaemia reflects a large sequestered parasite burden in falciparum malaria, and carries a worse prognosis [37]. By definition, patients with hyperparasitaemia represent the upper end of a frequency distribution of parasitaemia and so have more parasites in their blood than the majority of patients with malaria. If hyperparasitaemia is defined as >5% parasitaemia, then the number of blood stage parasites in the patient ranges between approximately 5 × 10^10 ^and 10^13 ^depending on body size and red cell count. Higher parasite burdens are impossible. Patients with uncomplicated malaria generally present with malaria at total body burdens between 5 × 10^6 ^and 10^10 ^parasites depending again on body size, and red cell count. Geometric mean parasite densities in drug trials of uncomplicated malaria range typically from 1000 to 20,000/uL blood. Parasite burdens in hyperparasitaemia, and the total number of cell divisions per infection, are therefore 10 to 10,000 times greater than in uncomplicated malaria. Thus, in terms of numbers of malaria parasites one hyperparasitaemic patient is equivalent to between 10 and 10,000 patients with symptomatic but otherwise uncomplicated malaria [3]. Thus, the number of mitoses at which resistance could have arisen is up to four orders of magnitude greater than in uncomplicated malaria and up to eight orders of magnitude greater than in parasites newly emergent from the liver stage development. Hyperparasitaemic patients also offer greater selection possibilities after the first drug exposed cycle as there are still up to 10^10 ^parasites present two days after starting treatment. Although selection probabilities are much lower (by a fraction of 1/PRR) in the second cycle (two days later) the drug levels may also be lower, and may now be within the window of selection (Figure 5). This is why hyperparasitaemic patients need longer courses of treatment with rapidly eliminated drugs.

#### Failure of host-defence

There is great variability in the severity of malaria infections. Even non-immune subjects infected with the same strain of parasite (as in the malariatherapy of neurosyphilis or in volunteer studies) varied considerably in the severity of infection that ensued [9-14]. Parasite clearance rates vary considerably between individuals infected with the same parasites and receiving similar anti-malarial treatments. The evolution of the human genome in tropical countries has been molded by the selective pressure of malaria. Hyperparasitaemia may, therefore, represent an unfortunate host-parasite combination in which unrestricted parasite growth is favoured. The development of hyperparasitaemia represents a failure of host-defence, both of the non-specific host-defence processes that normally limit parasite expansion and also the parasite specific immune responses that control parasitaemia in endemic areas. This indicates that an important obstacle to the emergence of resistance is lacking for the particular parasites (usually of a single dominant clone) causing the high parasitaemia.

#### Increased risk of recrudescence

**Figure 6:**
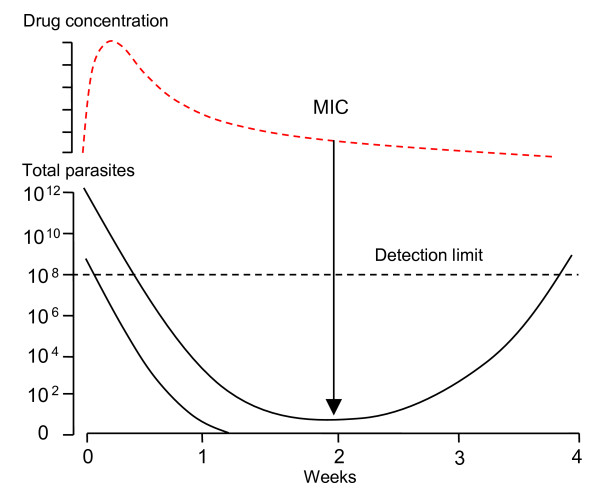
**Parasitaemia profiles in a recrudescent infection**. Patients with hyperparasitaemia are at greater risk of subsequent recrudescence even with fully sensitive parasites than patients with lower parasite densities. Each recrudescence provides an opportunity for enrichment (i.e. selection) of any resistant parasites formed. The total number of parasites in the body are shown on the lower vertical axis, drug concentrations of a slowly eliminated anti-malarial are shown in the upper panel. MIC: minimum inhibitory concentration.

Patients with hyperparasitaemia are invariably ill and, therefore, have a higher probability of being treated than other infections, and they are at considerably greater risk of failing treatment [27-31]. There are several reasons for this:

i) there are more parasites to eliminate and anti-malarial drug levels may fall below the minimum inhibitory concentration before the infection is extinguished (Figure 6).

ii) the patient is more ill, even if they do not yet have signs of severe malaria, and, therefore, they are more likely to vomit or malabsorb the anti-malarial treatment, thereby creating lower potentially selective blood concentrations. This may be particularly important, and is an argument for using an alternative route of drug administration, such as pre-referral rectal artesunate [38].

iii) hyperparasitaemic patients have by their nature "declared themselves" unable to control this particular infection.

Treatment failure means that retreatment is likely and provides a second round of selection, so that if the new resistant parasites are still in a minority (and contribute less than 10% of the gametocytes at this stage), they now have a second opportunity for selection, and have considerable parasite numbers present. This provides a greater chance of the resistant sub-population surviving "therapeutic concentrations" than in the original infection. Multiple recrudescences, therefore, provide a means of enrichment of resistant phenotypes.

### Implications

Hyperparasitaemic patients are an important source of de-novo resistance because of the larger numbers of parasites present, because the factors that led to these unusually heavy parasite burdens may impair the killing of de-novo resistant parasites, and because they have an increased risk of a subsequent recrudescent infection. Resistance is more likely to arise with high growth rates and high parasite burdens.

### Likely scenarios

For most resistance mechanisms, the initial level of resistance conferred is low, but with continued selection, the level tends to increase as further mutations are selected. The time scale is months to years. These new mutants can be resistance conferring, or compensatory mutations, which increase the fitness of resistant parasites. Selection of resistance in animal models is usually best achieved by giving a sub-curative dose, waiting for the parasite numbers to recover, and then repeating the dose [1,39-41]. This process may need to be repeated several times. This same process happens in clinical practice when an individual patient with hyperparasitaemia receives an inadequate single dose of anti-malarial, or vomits or malabsorbs the drug, and the full treatment course is not then received. These patients' infections are very likely to recrudesce. Some patients have unusual anti-malarial pharmacokinetics with expanded volumes of distribution and/or high drug clearance resulting in low blood concentrations. These are host properties and so these patients would be expected to have the same abnormalities on retreatment. This is a particular problem when dose regimens are relatively low (Table 2). It is worth noting that combination drugs still provide some protection against resistance in these scenarios [42].

**Table 2:** Drugs for which currently recommended anti-malarial regimens maybe inadequate in important patient groups.

**Drug**	**Dose**	**Patient group**	**Comment**
Dihydroartemisinin-piperaquine	2.5/20 mg/kg/day for 3 days	All	The current adult DHA dose of 120 mg may be too low. The piperaquine dose may be too low in children
Sulphadoxine -pyrimethamine	1.25/25 mg/kg	Children, pregnant women	Higher doses have not been evaluated
Atovaquone-proguanil	8/20 mg/kg for 3 days	Pregnant women	Higher doses have not been evaluated
Artemether-lumefantrine	1.5/9 mg/kg for 3 days with fat	Pregnant women Hyperparasitaemic patients	Lumefantrine absorption is dose limited. Longer courses have not been evaluated in pregnancy
Artesunate	Oral 2 mg/kg/day 7 days 2.4 mg/kg i.v.	Pregnant women	Higher doses have not been evaluated in pregnancy
Artemether	Oral 2 mg/kg/day 7 days 3.2 mg/kg i.m.	Pregnant women	Higher doses have not been evaluated in pregnancy

The efficacy of three day artemisinin-combination regimens in hyperparasitaemia is uncertain. Longer regimens may be necessary

A patient with fatal hyperparasitaemic falciparum malaria usually dies quickly and is, therefore, unlikely to transmit resistant parasites. The severely ill patient who survives will probably receive parenteral treatment, followed by a full course of oral treatment (now usually an ACT). This longer course of treatment will increase the chance of cure. The patient with "uncomplicated hyperparasitaemia" (i.e. with no evident clinical signs of severe malaria) who receives anti-malarial treatment as an outpatient, therefore, provides the greatest risk of selecting de-novo resistance as such patients usually receive no special attention and no special treatment. Identifying such patients and ensuring they receive adequate treatment would be expected to reduce the emergence of drug resistance.

#### Selecting artemisinin resistance

Artemisinins provide low resistance selection probabilities because they are eliminated very rapidly with a half-life of one hour or less. Thus there is almost no exposure to residual concentrations in the asexual cycle after the drug was taken. This creates a unique situation with regard to anti-malarial resistance selection. All other anti-malarials have some effects in more than one asexual cycle. In order for the artemisinin resistant parasites to survive and transmit the numbers of sensitive parasites must be reduced to less than ten times the number of resistant parasites. As the modal number of de-novo resistant parasites at initial drug exposure is one, this means reducing the number of sensitive parasites to <10. As a symptomatic infection always has >10^7 ^parasites, and as even the most artemisinin resistant parasites in the world are still killed by therapeutic levels of the drugs (with PRRs >10^2^), a single therapeutic dose of artemisinin (e.g. 4 mg/kg artesunate or artemether) cannot select for resistance, except in the unusual event described previously that relatively large numbers of de-novo artemisinin resistant parasites are present already because they arose at an earlier cell division [28,29].

#### Single dose exposure to therapeutic concentrations

**Figure 7:**
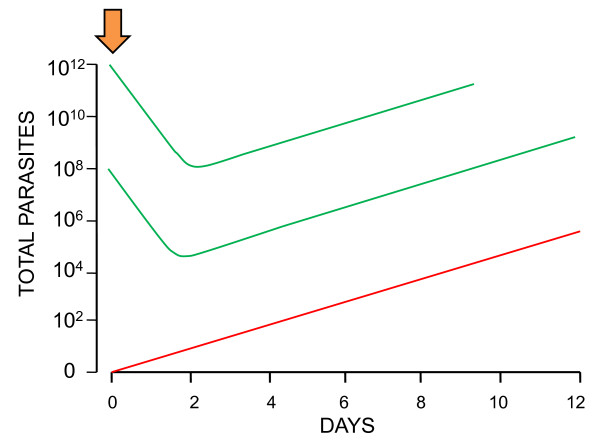
**Single dose exposure to an artemisinin (arrow) results in a reduction in the number of sensitive parasites by a factor (the PRR) of 10,000 fold**. But this is not enough to give a de-novo resistant parasites (red line) any chance of generating sufficient gametocytes for transmission as the sensitive sibling parasites (green lines) remain well ahead in the "race" to transmit.

If in uncomplicated malaria an adult with a low level infection of 10^8 ^parasites self-treats with a single treatment dose of artemisinin, then there will be 10^8 ^× 10^-4 ^= 10^4 ^parasites present in the next cycle one to two days later (in which there is no drug exposure). Even with no fitness disadvantage, there would need to be approximately 10^3^resistant parasites at this stage to transmit resistance (i.e. to comprise 10% of the subsequent gametocytes) as both resistant and sensitive parasites would then grow together at the same rate (assuming no second cycle effect and equal fitness) (Figure 7). If the first level of artemisinin resistance is a 99% reduction in killing efficacy (i.e. PRRs reducing from 10^4 ^to 10^2 ^per cycle), then there would need to have been 10^5 ^resistant parasites present in the previous cycle to generate 10^3 ^resistant parasites in the first post exposure cycle. At multiplication rates of 10 per cycle it is not possible to generate 10^5^resistant parasites from de-novo resistance emergence in the blood stage parasites. This can result only from de-novo emergence during liver stage development - an extremely rare event [6]. Only when total numbers of parasites in the body exceed 10^9 ^can this arise from a mitotic event in the blood stage parasites. For transmission of resistance, the final number of resistant gametocytes must eventually both exceed 10^7 ^and comprise ≥ 10% of the total gametocytes.

Assuming that one parasite initially is resistant then after *s *generations (binary divisions), there are 2^*s *^parasites. Each asexual cycle usually comprises five sets of divisions (from equation 3). If the PRR following artemisinin exposure is 10,000 and resistance reduces this by a factor *b *then after exposure to artemisinin there are 2^*s *^(*b*/10,000) resistant parasites

If the total number of (sensitive) parasites at the time when resistance first arises is C, then the corresponding number of sensitive parasites after a single artemisinin exposure is C^*s *^(1/10,000) sensitive parasites

The minimum conditions for transmission (assuming no fitness disadvantage) are therefore(1)

Likely values for b initially are less than 100, which suggests that artemisinin resistance could spread only in those rare instances in which resistance arose de-novo within the first 1,000 mitotic divisions of the liver stage development. Thus if 10^8 ^parasites are the minimum required to generate a transmissible density of gametocytes, only one of every 100,000 to 1,000,000 infections in which this level of artemisinin resistance arose de-novo, and were exposed to a single therapeutic dose of drug, could generate transmissible resistance. This presumably explains in part the rarity of de-novo artemisinin resistance emergence.

#### Single exposure to sub-therapeutic concentrations

Sub-therapeutic concentrations provide the opportunity for survival of much greater numbers of both sensitive and resistant parasites. But transmission of artemisinin resistance would require suppression of the sensitive parasites through repeated administration of artemisinin. The resistance risk for a single oral or rectal administration is therefore extremely low. Occasionally patients will take artemisinin or its derivatives for fever, which is not malaria, but by chance at the time of taking the drug merozoites are emerging from the liver. Up to 100,000 parasites may emerge and be exposed to artemisinin concentrations, but it is unlikely each hepatic schizont would mature at exactly the same time, and so the concentrations which each schizonts' progeny are exposed to will differ widely. This reduces the probability of selection by increasing the chance that some parasites will enter the blood after artemisinin concentrations have declined below therapeutic levels. In the example given above, where artemisinin resistance reduces PRRs 100 fold (from 10,000 to 100), the probability of resistance emergence exceeds 0.5 only if the following criteria are met:

1.) Hepatic schizont-derived merozoites (i.e. the first blood stage cycle) are exposed to drug concentrations in the window of selection (synchronous schizogony).

2.) The number of artemisinin resistant parasites exceeds 50. If each mature hepatic schizont has 30,000 merozoites (2^15^) then approximately 10% of all de-novo events per schizont would produce more than 50 parasites.

3.) The total number of blood stage parasites exposed to the drug is less than 100,000.

#### Multiple exposure to sub-therapeutic concentrations

In the case of artemisinins either systematic underdosing, intermittent self-medication with monotherapies, use of a sub-standard drug, or repeated administration of a rectal formulation without follow-up treatment provide the most likely contemporary scenarios for the de-novo emergence of resistance. This is because of the very low probabilities described above for resistance to emerge in a single in-vivo "selective sweep". Unfortunately the biological, pharmacological, operational, and behavioural factors which predispose to the first selection also predispose to the second and subsequent rounds of selection. A high parasitaemia provides an increased risk of recrudescence, and this predisposes to a second recrudescence even with observed treatment. Underdosing because of incorrect dose recommendations, poor adherence, or sub-standard drugs is likely to be repeated

### Implications

1. Inadequate dosing of uncomplicated hyperparasitaemia provides the most likely scenario for the de-novo emergence and selection of resistance.

2. Artemisinin resistance is very unlikely to be selected by a single exposure. Repeated inadequate dosing is required for selection and spread of artemisinin resistance.

## Discussion

Proving the exact individual origins of anti-malarial drug resistance in malaria endemic areas is probably impossible. To examine likely scenarios and guide prevention strategies induction from experimental observations and biological understanding is, therefore, combined with predictive mathematical modelling. Experimental evidence in laboratory animals and humans suggests that use of inadequate anti-malarial treatment doses, particularly in patients with hyperparasitaemia, may be an important source of de-novo resistance. It is both hyperparasitaemia itself (reflecting an unusually large number of parasites within a single host), and the host and parasite factors that gave rise to that hyperparasitaemia, which make it such a potentially dangerous source of anti-malarial drug resistance. These factors together increase the chance that a de-novo resistant parasite will emerge, and that its progeny will survive, that the infection will recrudesce, and that resistance bearing gametes will be transmitted.

Stable resistance can arise whenever there is nuclear division, so it may arise in patients with lower parasite burdens, as in uncontrolled self-medication, or from the exposure of the relatively small numbers of parasites emerging following hepatic schizogony. The dynamics of resistance emergence in low parasite biomass infections are generally similar to that in hyperparasitaemia but, even though an individual parasite's chance of survival may be higher, parasite numbers are orders of magnitude lower. Furthermore, the per-patient probability of an effective host response is greater in low biomass infections (in that hyperparasitaemia reflects an unusually poor host response and or an unusually favourable host for that particular parasite). Asymptomatic parasitaemia reflects an effective density controlling host response, and as a consequence a low individual probability that any newly emergent resistant parasites could generate transmissible gametocyte densities, even if anti-malarial treatment was taken for an incidental fever. An adult with 10% parasitaemia contains approximately the same number of parasites as 10,000 people with aymptomatic parasitaemia. Low parasite biomass infections are therefore an unlikely source of de-novo resistance.

The importance of residual drug levels from previous anti-malarial treatments exposing newly acquired infections to sub-therapeutic selective drug concentrations has been much debated as a possible source of resistance [43]. Slowly eliminated, "long half-life" anti-malarials have been considered particularly vulnerable because they present a gradient of concentrations over an extended time period, and therefore a protracted selection opportunity [34]. This is very important as a selective force in the spread of resistance, but there are several reasons why this is not an important determinant of de-novo selection. The first is numerical. The numbers of parasites emerging from the liver (circa 10,000 to 100,000), and thus the number of mitoses which could give rise to a resistant parasite, are over one thousand times lower than in symptomatic malaria, and approximately one hundred million fold lower than in a hyperparasitaemic patient [3]. The second factor is "immunity". In high transmission settings people are bitten frequently and also take large quantities of anti-malarial drugs so new infections will commonly "see" sub-therapeutic levels of anti-malarial drugs. This provides a de-novo selection opportunity, but survival probabilities for the newly emergent resistant parasite's progeny are low. They must evade the primed host defences and outcompete the usually much larger numbers of sensitive parasites from the same infection and others subsequently acquired in the race to produce gametocytes. A single resistant parasite's progeny would be expected to take 8-20 days longer than its 10^4 ^to 10^5 ^drug sensitive siblings to generate transmissible gametocyte densities with unrestricted multiplication. Most acquired infections in high transmission settings do not generate transmissible gametocyte densities because of effective host defence, so the chances of a de-novo resistant parasite avoiding host defence mechanisms for an additional one to three weeks are likely to be very low. For artemisinin and its derivatives, there is usually no possibility of selecting resistance in liver-emergent blood stage parasites because the drugs are rapidly eliminated from the body and because of competition from residual drug sensitive sibling parasites [28]. A third factor in areas of stable malaria transmission, which may be relevant to some resistance mechanisms, is that multiplicity of infection increases the chance of recombination breakdown of multigenic resistance.

To illustrate further the numerical differences between hyperparasitaemia and exposure of newly acquired infections to residual drug levels, an area where each person receives on average 10 infectious bites each year (EIR 10) can be considered. It would take one million people ten years to accumulate the same number of hepatic emergent parasites (and thus mitotic events) as in one hyperparasitaemic individual. Each of these newly emergent infections offers a low individual probability of surviving to transmit, and an even lower probability that a de-novo resistant parasite within the infection could multiply enough to generate transmissible gametocyte densities. In contrast, if the single hyperparasitaemic individual receives inadequate treatment, recrudescence (and thus an opportunity for selection) is almost inevitable if the patient survives. The odds of resistance generation are therefore heavily stacked towards the hyperparasitaemic patient. Even so the individual probabilities of a resistant parasite surviving and its progeny being transmitted subsequently are very low unless either there are large numbers of resistant parasites (having emerged earlier in development) (Figure 3), or the new resistance mechanism allows survival in the presence of therapeutic drug concentrations (high grade resistance).

Previous assessments of resistance have tended either to consider resistance as a binary (fully resistant/fully sensitive) or ternary (resistant, tolerant, sensitive) process, and then mainly in the context of resistance spread [44]. The degree of resistance conferred by the genetic event is critical in determining the probability of de-novo selection. Selection of low levels of resistance usually requires multiple recrudescences before the resistant parasites predominate and can transmit. Unfortunately multiple recrudescences are common, particularly in low transmission settings. Once one recrudescence has occurred then for one or more reasons (adherence, drug quality, pharmacokinetics, immunity) the host- parasite combination is unusually favourable for that particular infection.

Molecular epidemiological studies have provided important information on the relative importance of de-novo emergence and spread in the global increase in anti-malarial drug resistance. It is now evident that spread of resistance is more important than originally thought, and that the predominant anti-malarial drug resistance genotypes derive mainly from relatively few de-novo events. Despite extensive use of sulphadoxine-pyrimethamine in high transmission areas of Africa over the past two decades, and prior use of pyrimethamine alone, these epidemiological studies indicate that the high level resistance that has spread across Eastern and Central Africa arose in Asia [45]. The spread of chloroquine resistance preceded it along the same route. Most evidence points to low transmission areas as the main source of resistance. In low transmission settings where individual parasites have a better chance of surviving in the largely non-immune human hosts, treatment is used less frequently. As a result, the probability that liver-emergent parasites would encounter residual sub-therapeutic anti-malarial concentrations from a previous drug administration is reduced. Even if they do, and are not recognized initially by the host-immune response, any resistant mutant arising must outcompete its majority drug- sensitive siblings in the race to produce gametocytes or fail any subsequent treatment. The "advantage" that a primary infection has over a recrudescent infection of not having provoked a specific immune response is offset by all these other factors.

The importance of correct anti-malarial dosing in the prevention of resistance has not received sufficient attention. Unfortunately, systematic underdosing of those patients with the most parasites and the least immunity (children, pregnant women, hyperparasitaemics) has been common, as anti-malarial dose regimens have been extrapolated from studies in adults and have often recommended doses which are too low [36]. Initial dose recommendations for sulphadoxine-pyrimethamine in children and for mefloquine were inadequate, and may well have engendered resistance [36,46,47]. Initial dose regimens of artemether-lumefantrine were also too low. Pregnant women may be an important source of resistance. They may have a considerable burden of parasites in the placenta, have poor host-defence against malaria and usually have lower anti-malarial drug levels than non-pregnant adults. Some patients have unusual pharmacokinetics resulting in low drug levels. This is a host property so on retreatment they will usually have low levels again, contributing to the risk of recrudescence and resistance selection. Anti-malarial dose regimens should be designed to achieve effective drug concentration profiles in nearly all patients. Many do not (Table 2).

Hyperparasitaemia is an important source of resistance but it is often not recognized. Most patients with falciparum malaria do not obtain a diagnosis, and of those who do get parasitological assessment, few have accurate counts made. Rapid diagnostic tests are being introduced but are used qualitatively not quantitatively. It was once recommended that children with a fever in high transmission areas should receive anti-malarial drugs without making a diagnosis. This position is now changing. If patients with hyperparasitaemia could be identified, and were given an extended course of curative treatment, the emergence of anti-malarial drug resistance might be delayed. These observations emphasize the importance of ensuring that anti-malarial drug quality is good, that monotherapies are never used, that doses are optimized on the basis of pharmacodynamic and pharmacokinetic assessment in the target populations, and that patients with heavy parasite burdens receive adequate treatment to prevent recrudescence.

## Abbreviations

PMF: parasite multiplication factor; PRR: parasite reduction ratio; IC: inhibitory concentration; E: parasite blood stage multiplication efficiency; *mdr1*: genes encoding the parasite multidrug resistance transporter; *dhfr*: genes encoding the parasite dihydrofolate reductase; *dhps*: genes encoding the parasite dihydropteroate synthase; MPC: minimum parasiticidal concentration; MIC: minimum inhibitory concentration; ACT: artemisinin combination treatment.

## Competing interests

The authors declare that they have no competing interests.

## Authors' contributions

NW conceived the study and conducted the analysis and interpretation and drafted the manuscript. WP, RM, SS, RA, KS, SL, AD, LW, ND contributed to the analysis and interpretation, and reviewed the manuscript. All authors read and approved the final manuscript.

## Supplementary Material

Additional file 1 **Antimalarial drug resistance emergence probabilities**. An examination of the probabilities of resistance emergence in relation to parasite multiplication rates of resistant and sensitive parasites and total parasite burdens. Click here for file

Additional file 2 **Pharmacokinetics and pharmacodynamics**. An examination of the relationship between the pharmacokinetic properties of the antimalarial drugs, their blood stage pharmacodynamic properties, and the anti-malarial drug resistance selection opportunities and probabilities. Click here for file
