# Supplementary material for: Hyperparasitaemia and low dosing are an important source of anti-malarial drug resistance
Source: Malar J. 2009 Nov 11;8:253. doi: 10.1186/1475-2875-8-253 (PMC2784792; doi:10.1186/1475-2875-8-253)
Supplement: Additional file 1 — Antimalarial drug resistance emergence probabilities. An examination of the probabilities of resistance emergence in relation to parasite multiplication rates of resistant and sensitive parasites and total parasite burdens. [file 1475-2875-8-253-S1.doc]

**Additional file 1**

**Antimalarial drug resistance emergence probabilities**

Here the probabilities of resistance emergence in relation to parasite multiplication rates of resistant and sensitive parasites and total parasite burdens are examined . As multiplication is the sum of a series of binary divisions the probability distributions are binomial.

1. ***Events within the blood stage schizont***

The probability (p*_q_*) that a parasite mutated during *q* generations of binary division is:

p*_q_* = 1 – (1 – p)*^q^* ……..……………………………………………………...….(1)

where p is the probability of mutation during a single binary division.

Hepatic and blood stage schizonts are formed by a series of mitotic binary divisions. Starting with one parasite, x series of mitotic divisions (successive series of 1, 2, 4, 8 …,2^X-1^ divisions) would result 2^X^ merozoites. Therefore the total number of binary divisions (N) to form a schizont is given by the number of merozoites (M) minus one.

N= M-1……………………………………………………………………….(2)

For example if the average total number of merozoites per blood stage schizont (M) is 32 then this represents 5 series of mitotic divisions (2^x^ where x = 5). The number of actual divisions (M-1) is 31 corresponding to 1+2+4+8+16 divisions. If there is no loss of parasites and full reproductive efficiency then a liver schizont of approximately 30,000 merozoites results from 15 series of mitotic divisions and approximately 30,000 binary divisions. Resistance could have arisen in any of these steps. Obviously if it occurred at an early step it would result in more than one resistant progeny. If we assume the probability of mutation is the same among the mitotic divisions, then if resistance does occur de-novo during the formation of the blood-stage schizont, the probability of there being one resistant merozoite is 16/31 (52%) where the resistance mutation occurred in the last series of mitotic divisions, of two resistant merozoites is 8/31 (26%), of four is 4/31 (13%), of eight is 2/31 (6 %) and of 16 is 1/31 (3%).

In the absence of drugs the ratio of F, the parasite multiplication factor to the average number of merozoites per mature schizont (M) can be termed E, the parasite multiplication efficiency

Thus E = F/ M……………………….………………………………………....….(3)

E can start with values exceeding 0.5, but it falls abruptly as high parasite densities are reached as a result of several factors (fever, reduced susceptible red cell availability, increased host-defence, quorum sensing). Obviously anti-malarial drug treatment reduces parasite multiplication. The differential effects on multiplication are analysed further in Additional file 2.

Before assessing drug effects it is necessary to describe merozoite survival probabilities in the absence of drugs. The probability of resistance (*p_r_)* surviving to the next asexual generation is

*p_r_*  = 1- (1-E)*^r^* …………………….…….…… …………………………………….(4)

where *r* is the number of resistance-bearing merozoites in a schizont.

Therefore if resistance arose spontaneously during schizogony then the probability (*p_r_*) of an individual parasite’s progeny surviving to the next cycle is E. Occasionally a schizont will contain more than one resistant parasite if resistance arose at an early binary division.

Thus the overall probability of one or more parasites surviving (*p_R_*) if resistance arose in a schizont is the sum of the products of the individual probabilities of emergence and survival. From equations 2. and 4. then for schizonts with M merozoites this gives;

*p_R_* = ∑{[1-(1-E)*^r^* ][ (M/2*r*) /(M-1)]} …………………………………..……….(5)

for r = 1, 2, 4, 8 or 16. If the corresponding probabilities (p_r_) for these r values are p_1_ to p_5_  then in the example adding all the individual probabilities together for the numbers of resistant merozoites for schizonts with 32 merozoites gives:

16/31(p_1_) + 8/31(p_2_) + 4/31 (p_3_) + 2/31 (p_4_) + 1/31 (p_5_)…………………………...(6)

Thus if resistance arose in a schizont, the overall probabilities that a resistance bearing merozoite would invade a new erythrocyte at multiplication efficiencies (E) of 0.3, 0.4, and 0.5 would be 0.48, 0.58 and 0.67 respectively. The drug resistance mechanism often impairs parasite fitness (*f*), and thus reduces the multiplication factor compared with that of sensitive parasites. This can be expressed as the ratio of growth rates (F) of resistant and sensitive parasites;

*f* = F_R_/ F_S_…………………………………………………………………………..(7)

Multiplication efficiency (E) for the drug resistant parasites (E*_R_*) in the absence of drugs is therefore *f*  E*_S_* where E*_S_* is the multiplication efficiency of the predominant sensitive parasite population.
