# Supplementary material for: Hyperparasitaemia and low dosing are an important source of anti-malarial drug resistance
Source: Malar J. 2009 Nov 11;8:253. doi: 10.1186/1475-2875-8-253 (PMC2784792; doi:10.1186/1475-2875-8-253)
Supplement: Additional file 2 — Pharmacokinetics and pharmacodynamics. An examination of the relationship between the pharmacokinetic properties of the antimalarial drugs, their blood stage pharmacodynamic properties, and the anti-malarial drug resistance selection opportunities and probabilities. [file 1475-2875-8-253-S2.doc]

**Additional file 2**

**Pharmacokinetics and pharmacodynamics** [18,33]

Peak anti-malarial drug concentrations occur in the blood during the first drug exposed cycle if a single dose is given. If multiple doses are given, then for all drugs except artemisinins and quinine, the peak concentrations will follow the last dose.

The drugs are eliminated by first order processes which can be characterized by one or more exponential terms. The simplest is an open “one compartment model”

C(t) = C(0) * exp^(-kt)^………………………………………………(1a),

where k=log_e_2 / t_1/2_ is the terminal elimination rate constant, t is time and C(t) is concentration at time t, and C(0) is the maximum concentration. Multiexponential models are often required for adequate fitting of concentration-time data.

The relationship between parasite killing and the drug concentration C (concentration-effect or dose-response relationship) is sigmoid and can be described by a sigmoid Emax model :

f(C ) = - k_1_ • C^n^ / (C^n^ + EC50^n^)………………………………...… (2a)

where k_1_ is the first order rate constant for maximum parasite killing, n is the slope the concentration-effect curve, C is the anti-malarial drug concentration, and EC_50_ is the drug concentration which kills 50% of the parasites which could have been killed if the drug had maximum effect (Emax). For example the k_1_ for a drug with a PRR of 1000/cycle would be ln(10^-3^)/2.

We assume that the shape of the concentration-effect relationship and its slope are similar in-vitro and in-vivo, even if the absolute values for other parameters are shifted, and for simplicity assume that the general shape of the concentration- effect relationship is the same in the susceptible and resistant populations (i.e. the same slope ***n*** and same maximum effect). Resistance reflects then a parallel shift to the right in the resistant population, and the degree of resistance is measured by the ratio of respective EC50’s *(R);*

*R* = EC50 (resistant) / EC50(sensitive). ………….………………… (3a)

More complex changes in shape of the concentration-effect relationship with resistance could be incorporated.

The mathematical relationship between the changes in total numbers of parasites and time, in the presence of anti-malarial drug, was described by Simpson et al (36):

EC50 *^n^* + C(0)*^n^* e –***^n^*** ^k t^

P(t) = P(0) • e ^k^_2_^•t^ • [ ────────── ] ^-k^_1_^/(k^ ***^n)^*** ……..…………… (4a)

EC50 *^n^* + C(0)*^n^*

where P(t) is total number of parasites or parasite biomass at time t>0,

and P(0) is the total number of parasites at time t=0,

k_2_ = ln(E x M)/2 is first order rate constant of maximum parasite multiplication rate (per day) , C(0), EC50, k, k_1_ and *n* are defined as before. Maximum parasite multiplication (E x M) usually varies between 5 and 20 per two day cycle

So if the blood stage infection starts with P_s_(t_0_ ) sensitive parasites and P_r_(t_0_ ) resistant parasites, then at time t we will have P_s_(t) sensitive parasites and P_r_(t ) resistant parasites as shown:

1 + ***a****^n^* e –***^n^*** ^k t^

P_s_(t) = P_s_(t_0_) • e ^k^_2_^•(t)^ • [ ───────── ] ^-k^_1_^/(k^ ***^n)^***

1+***a****^n^* ………………………(5a)

*R^n^* + ***a****^n^* e –***^n^*** ^k t^

P_r_(t) = P_r_(t_0_) • e ^k^_2_^•(t)^ • [ ────────── ] ^-k^_1_^/(k^ ***^n)^***

*R^n^* +***a****^n^* ……………………….(6a)

where ***a*** is the ratio between C(0) and EC50, ***p*** is the ratio of EC50’s for resistant and sensitive parasites, and k, k_1_, k_2_ and *n* are defined as before.

There are four parameters which can be used to characterize the pharmacokinetic/pharmacodynamic properties of the anti-malarial drug :

1. the terminal elimination half life (***t_1/2_*** ) which is related to the terminal elimination rate constant

k = ln(2)/ ***t_1/2_***

1. the ratio of the maximum drug concentration (at time 0), C(0) and EC50 - ***a*.**
2. the slope of the linear portion of the concentration-effect relationship – ***n*.**

4. the ratio of EC50’s for resistant and sensitive parasites ***R***
